# IgSF11 deficiency alleviates osteoarthritis in mice by suppressing early subchondral bone changes

**DOI:** 10.1038/s12276-023-01126-6

**Published:** 2023-12-01

**Authors:** Gyeong Min Kim, Jihee Kim, June-Yong Lee, Min-Chan Park, Soo Young Lee

**Affiliations:** 1 Department of Life Sciences, Ewha Womans University, Seoul, 03760 Republic of Korea; 2 The Research Center for Cellular Homeostasis, Ewha Womans University, Seoul, 03760 Republic of Korea; 3 Department of Microbiology and Immunology, Institute for Immunology and Immunological Diseases, and Brain Korea 21 PLUS Project for Medical Sciences, Yonsei University College of Medicine, Seoul, 03722 Republic of Korea; 4 Division of Rheumatology, Department of Internal Medicine, Yonsei University College of Medicine, Seoul, 06273 Republic of Korea; 5 Multitasking Macrophage Research Center, Ewha Womans University, Seoul, 03760 Republic of Korea

**Keywords:** Osteoarthritis, Bone

## Abstract

Osteoarthritis (OA) is a degenerative joint disease. While it is classically characterized by articular cartilage destruction, OA affects all tissues in the joints and is thus also accompanied by local inflammation, subchondral bone changes, and persistent pain. However, our understanding of the underlying subchondral bone dynamics during OA progression is poor. Here, we demonstrate the contribution of immunoglobulin superfamily 11 (IgSF11) to OA subchondral bone remodeling by using a murine model. In particular, IgSF11 was quickly expressed by differentiating osteoclasts and upregulated in subchondral bone soon after destabilization-of-the-medial-meniscus (DMM)-induced OA. In mice, IgSF11 deficiency not only suppressed subchondral bone changes in OA but also blocked cartilage destruction. The IgSF11-expressing cells in OA subchondral bone were found to be involved in osteoclast maturation and bone resorption and colocalized with receptor-activator of nuclear-factor κ-B (RANK), the key osteoclast differentiation factor. Thus, our study shows that blocking early subchondral bone changes in OA can ameliorate articular cartilage destruction in OA.

## IgSF11: the game-changer in osteoarthritis subchondral bone remodeling

Osteoarthritis (OA, a common joint disease in adults due to imbalance in cartilage formation and decay), is a complex process impacting the entire joint with no current treatments. A research team led by G.M.K and S.Y.L looked into the function of Immunoglobulin superfamily-11 (IgSF11, a protein aiding cell sticking together and osteoclast differentiation - a process creating a type of bone cell). This protein is rapidly produced in osteoclasts undergoing differentiation and in the osteoarthritic subchondral bone (bone layer beneath joint cartilage). Removing IgSF11 in mice resulted in less osteoclast activity, decreased bone thickening, and reduced cartilage breakdown in OA. The researchers believe that aiming at osteoclasts in subchondral bone could potentially treat OA effectively.

This summary was initially drafted using artificial intelligence, then revised and fact-checked by the author.

## Introduction

Osteoarthritis (OA) is the most prevalent joint disease in adults, and its incidence is rising annually. Although this disease is disabling and reduces quality of life, OA disease-modifying treatments are lacking^1,2^. OA is generally thought to be largely due to dysregulated articular cartilage formation/resorption that promotes cartilage degeneration^3,4^. However, OA also involves subchondral bone (SB) sclerosis and inflammation in the synovium^5–8^. Indeed, it is now increasingly clear that OA is a complex multifactorial disease that affects the whole joint and that the various joint changes all interact with each other to promote OA development and progression^9,10^.

The initial pathological changes that drive OA remain unclear. However, it is increasingly suspected that changes in the SB underlying the cartilage play key early and ongoing roles^11^. The SB, which provides the cartilage with structural and nutritional support, is acutely responsive to mechanical forces on the cartilage: these forces rapidly reshape the balance between SB formation and resorption, which are mediated by osteoblasts and osteoclasts, respectively. In OA, the SB balance is strongly disrupted, resulting initially in osteoclast formation and increased bone resorption. Later, the empty spaces activate osteoblasts, which fill the spaces with bone in a process called sclerosis^12,13^. These SB changes directly influence the cartilage. Thus, bone resorption destabilizes the cartilage mechanically, which induces chondrocyte apoptosis. Early osteoclasts also shift chondrocyte function toward cartilage resorption by releasing TGF-β and other bioactive molecules and by migrating into cartilage and physically contacting chondrocytes. Since blocking osteoclast formation and function can attenuate OA progression and SB remodeling in OA is closely related to angiogenesis, nerve growth, and pain^11,13^, interest in therapeutically targeting osteoclasts and SB sclerosis has grown over recent years. However, this objective is hampered by our poor understanding of the molecular mechanisms that underlie SB dynamics.

Immunoglobulin superfamily 11 (IgSF11) (also known as BT-IgSF11) is a member of the immunoglobulin superfamily. This molecule has two extracellular immunoglobulin-like domains and a cytoplasmic region bearing the PDZ-binding motif. IgSF11 was first identified as a cell-adhesion molecule that mediates homophilic interactions between cells in a calcium-independent manner^14,15^. This molecule is expressed in the brain, where it regulates synaptic transmission and plasticity by acting as a synaptic-adhesion molecule; this function is mediated by interactions with the postsynaptic scaffolding protein PSD95, which has a PDZ-binding domain^16^. IgSF11 is also expressed in the testis and promotes male fertility by maintaining the blood‒testis barrier^17^. Moreover, IgSF11 inhibits human T cells by binding to the V-domain Ig-suppressor of T-cell activation^18^. Notably, recent studies have shown that IgSF11 is expressed in osteoclast precursors, and IgSF11-PSD95 signaling is essential for the interosteoclast adhesion and fusion that induces their maturation in vitro^19,20^. IgSF11 also drives osteoclast formation by regulating pyruvate kinase M2 activity and cellular metabolism. Thus, IgSF11 is a vital activator of osteoclast differentiation and function. Interestingly, in healthy mice, excessive and impaired IgSF11-PSD-95 signaling induces osteoclastic bone loss and increased bone mass, respectively^19,20^.

These observations suggest that IgSF11 could participate in OA. This notion was tested in the present study. Indeed, we found that IgSF11 is expressed by differentiating osteoclasts and that IgSF11^+^ osteoclast-like cells emerge in the SB soon after OA induction. Moreover, we found that IgSF11 deletion attenuated both early SB remodeling and subsequent articular cartilage destruction. Thus, targeting osteoclasts in the SB may be a promising therapeutic strategy for OA.

## Materials and methods

### In vitro osteoclast differentiation

Bone marrow-derived macrophages (BMMs) were generated from bone marrow from 4- to 6-week-old wild-type (WT) C57BL/6 mice (Jackson Laboratory, USA) or *IgSF11*-deficient (*IgSF11*^*−/−*^) mice (provided by Eunjoon Kim (Korea Advanced Institute of Science and Technology, South Korea) by culture in alpha-minimum essential medium (α-MEM; HyClone) containing 10% fetal bovine serum (HyClone) and 1% penicillin/streptomycin (Gibco, USA) for 24 h. Nonadherent cells were then incubated with 30 ng/ml macrophage colony-stimulating factor (M-CSF; R&D Systems) and 100 ng/ml receptor activator of nuclear factor κ-B (RANK) ligand (RANKL; R&D Systems) for 3 days.

### In vitro culture of primary articular chondrocytes with osteoclast-conditioned medium

For generation of osteoclast-conditioned medium (OC-CM), BMMs from WT and *IgSF11*^*−/−*^ mice were induced to differentiate into mature osteoclasts on dentin slices with M-CSF and RANKL for 4 days. OC-CM was collected on day 5 and stored at −80 °C. Primary articular chondrocytes were isolated from the femoral condyles and tibial plateaus of 4–5-day-old ICR mice by digestion with 0.2% collagenase type II. The chondrocytes were cultured for 24 h with or without OC-CM in Dulbecco’s modified Eagle’s medium (DMEM; HyClone, Logan, UT, USA) containing 10% fetal bovine serum.

### Experimental OA in mice

Male 10–12-week-old C57BL/6J and *IgSF11*^*−/−*^ mice were used. The mice were housed in a pathogen-free barrier facility at ≤5/cage at 24–26 °C with 30–61% humidity and 12 h light/dark cycles. After 1 week of adaptation, experimental OA was induced by destabilization of the medial meniscus (DMM) surgery. Thus, the medial-meniscus ligament of the right knee joint was surgically removed^21^. Sham-operated mice served as controls. After euthanasia at 1, 2, 4, or 8 weeks, the knee joint tissues were subjected to histology. For each experiment, mice were age- and sex-matched. All animal experiments were approved by the Institutional Animal Care and Use Committees (Protocol No: IACUC 23-036) of Ewha Womans University and followed National Research Council Guidelines.

### Human samples

Joint tissues were obtained from ten patients with OA during total knee replacement surgery. In all cases, the cartilage had an Osteoarthritis Research Society International (OARSI) score of 6. OARSI is a standard OA-grading system (grades 0–6)^22^. The patients were 63–78 years old (3 males and 7 females). For elimination of the effects of other underlying diseases, only OA patients without RA, metabolic diseases, or other inflammatory diseases at the time of surgery were included. The use of the joint tissues was approved by the Institutional Review Board of Gangnam Severance Hospital, Seoul, South Korea (IRB No: 3-2023-0252). All participants provided informed consent.

### Western blotting analysis

Cells were lysed in cell lysis buffer (50 nM Tris-HCl, pH 8.0, 150 mM NaCl, 0.5% deoxycholate acid, 1% NP-40) containing protease and phosphatase inhibitors. Western blotting was conducted with antibodies against IgSF11 (2067, 1:500) (provided by Eunjoon Kim of Korea Advanced Institute of Science and Technology, South Korea), anti-NFATc1 (Cat# sc-7294, 1:1000) and anti-β-actin (Cat# sc-47778, 1:1000).

### Histology and immunohistochemistry

The knee joints were fixed in 10% formaldehyde at 4 °C for >48 h, decalcified in 0.5 M EDTA (pH 7.4) for 14 days, embedded in paraffin, cut into 5-μm sections, and stained with safranin-O with fast green counterstaining. Articular cartilage destruction was scored using OARSI. The tartrate-resistant acid phosphatase (TRAP)-staining kit was from Fujifilm Wako Pure Chemical Corporation (Japan). For immunohistochemistry, knee joint sections were incubated overnight at 4 °C with antibodies against MMP3 (Cat# Ab53015, 1:50), MMP13 (Cat# Ab39012, 1:50), Aggrecan (Cat# Ab1031, 1:100), pSmad3 (Cat# Ab52903, 1:100), Osterix (Cat# Ab22552, 1:400) (all from Abcam), or COL2A1 (Cat# MAB8887, 1:100; Sigma-Aldrich). Immunoactivity was detected with a DAB peroxidase-substrate detection kit (Vector Laboratories, USA). Nuclei were counterstained with hematoxylin. Samples were measured using OsteoMeasureXP (OsteoMetrics, Inc., Atlanta, GA, USA), Adobe photoshop (v19.1.3), and an Olympus DP72 charge-coupled device camera (v2.1, Olympus Corporation, Tokyo, Japan).

### Immunofluorescence staining

Tissue samples were embedded in OCT and sectioned into 10-µm slices. Tissue slices were permeated with Triton X-100 (0.01%, Sigma), blocked in 5% horse serum, and incubated overnight at 4 °C with antibodies specific for IgSF11 (Cat#sc-393816, 1:100), colony-stimulating factor 1-receptor (CSF-1R) (Cat#sc-692, 1:50), RANK (Cat#22215, 1:100; Cell Signaling Technology), TRAP (Cat# 32694, 1:200; SAB), cathepsin K (CTSK) (Cat#ab19027, 1:100), and ATPase H+ Transporting V0 Subunit D2 (Atp6v0d2) (provided by Y. Choi, University of Pennsylvania, Philadelphia, PA, USA). The slides were then incubated at room temperature for 1 h in the dark with Alexa-Fluor Plus 594-rabbit (Cat#A32754), Alexa-Fluor Plus 594-mouse (Cat#A32744), Alexa-Fluor Plus 488-rabbit (Cat#A32790), and Alexa-Fluor Plus 488-mouse (Cat#A32766) (Thermo Fisher Scientific) and visualized under a Zeiss LSM880 Airyscan confocal microscope (Germany).

### RNA isolation and quantitative real-time PCR (qRT‒PCR)

Total RNAs from SB were isolated using TRIzol reagent (Invitrogen, USA), reverse-transcribed using the Superscript cDNA synthesis kit (Invitrogen), and subjected to real-time PCR with the KAPA SYBR Green fast qPCR kit (Kapa Biosystems, USA) on a Step One Plus RT‒PCR machine (Applied Biosystems, USA). The samples were analyzed in triplicate, and the data were normalized to β-actin-mRNA expression. The primers were as follows: IGSF11: forward: 5’-CGGTGCTGTTCTTATCGTCATC-3’, reverse: 5’-TTCTTCCTCCTCCTCCTCTTTGT-3’; RANK: forward: 5’-GGTTATGTAATGAGCGGCAGCA-3’, reverse: 5’-TTCTCATCGGCACTGTAGATCTGG-3’; MMP3: forward: 5’-TCCTGATGTTGGTGGCTTCAG-3’, reverse: 5’-TGTCTTGGCAAATCCGGTGTA-3’; MMP13: forward: 5’-CCTTGAACGTCATCATCAGG-3’, reverse: 5’-TGTTTATTGTTGCTGCCCAT-3’; COL2A1: forward: 5’-CACACTGGTAAGTGGGGCAAGA-3’, reverse: 5’-GGATTGTGTTGTTTCAGGGTTCG-3’; ACAN: forward: 5’-GAAGACGACATCACCATCCAG-3’, reverse: 5’-CTGTCTTTGTCACCCACACATG-3’; and ACTIN: forward: 5’-GCTTCTTTGCAGCTCCTTCGT-3’, reverse: 5’-ATCGTCATCCATGGCGAACT-3’.

### Quantitation and statistical analysis

All data were from ≥3 independent experiments. Two groups were compared by two-way ANOVA followed by multiple pairwise comparisons with Tukey’s test if significant. Sample sizes for each experiment were not predetermined. *p* values are indicated in the figures. Error bars represent standard error-of-the-mean (S.E.M.) for parametric data and 95% confidence intervals for nonparametric data. All graphs and statistical analyzes were generated with GraphPad Prism (v8.1.2).

## Results

### IgSF11 is quickly expressed in the SB in murine OA

**Fig. 1:**
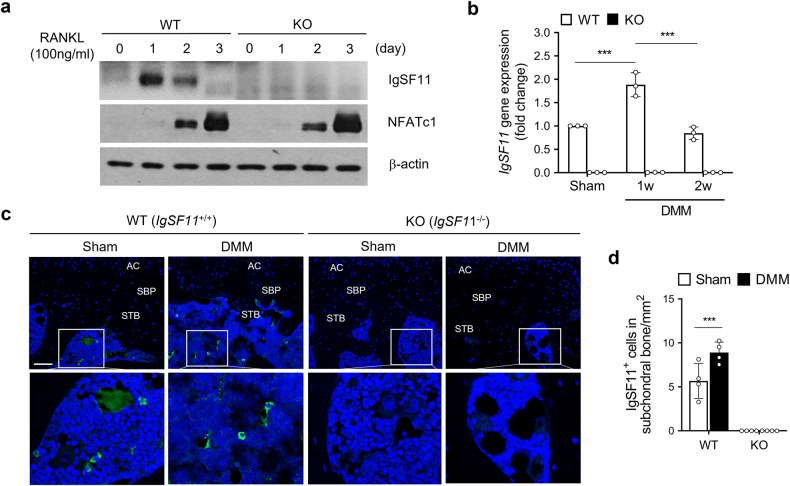
IgSF11 expression is quickly upregulated in vitro, differentiating osteoclasts and osteoarthritic subchondral bone. **a** BMMs from WT and *IgSF11*^*−/−*^ mice were induced to differentiate into mature osteoclasts by culture with M-CSF and RANKL for 3 days. The protein levels of IgSF11, the osteoclast activation-related protein NFATc1, and β-actin at 1, 2, and 3 days were detected by western blotting. **b**–**d** WT and *IgSF11*^−/−^ mice underwent DMM or sham surgery. **b** The subchondral bone was harvested at 1 or 2 weeks and subjected to qRT‒PCR analysis of IgSF11. **c**, **d** Whole joint sections were subjected to immunofluorescence analysis of IgSF11 (green) at 1 week. **c** Representative immunofluorescence images. AC articular cartilage, SBP subchondral bone plate, STB subchondral trabecular bone. Scale bars, 100 μm. **d** shows the IgSF11^+^ cells per bone marrow area (mm^2^). Error bars show the S.E.M. for *n* = 4/strain. Two-way ANOVA followed by Tukey’s multiple comparison test was conducted. *p* values are indicated in the figures (***p < 0.001).

Since recent studies have shown that IgSF11 is required for osteoclast maturation^19,20^, we first confirmed that osteoclastogenesis-inducing RANKL stimulation increased IgSF11 expression in BMMs. Indeed, IgSF11 protein expression rose, with a peak on day 1 (Fig. 1a). We then examined whether IgSF11 is expressed in the SB of mice 1 or 2 weeks after DMM surgery-induced OA. DMM is a widely used animal model for researching cartilage degradation and SB dynamics in OA^21,23^. qRT‒PCR of the SB from WT mice showed that IgSF11 mRNA expression rose in the SB at 1 week and then fell at 2 weeks (Fig. 1b). Thus, IgSF11 is quickly upregulated in the SB after OA induction.

Notably, chondrocytes do not express IgSF11; immunofluorescence analysis of the joint tissues of WT mice with and without DMM surgery-induced OA showed that if IgSF11 was expressed, it was always in the SB and not the cartilage (Fig. 1c, d). This phenomenon was also observed when we conducted immunohistochemical analyses of damaged and undamaged cartilage from OA patients undergoing total knee replacement surgery (Supplementary Fig. 1). Thus, the expression of IgSF11 in OA is limited to the SB.

### Genetic *IgSF11* deletion attenuates articular cartilage degeneration in OA

**Fig. 2:**
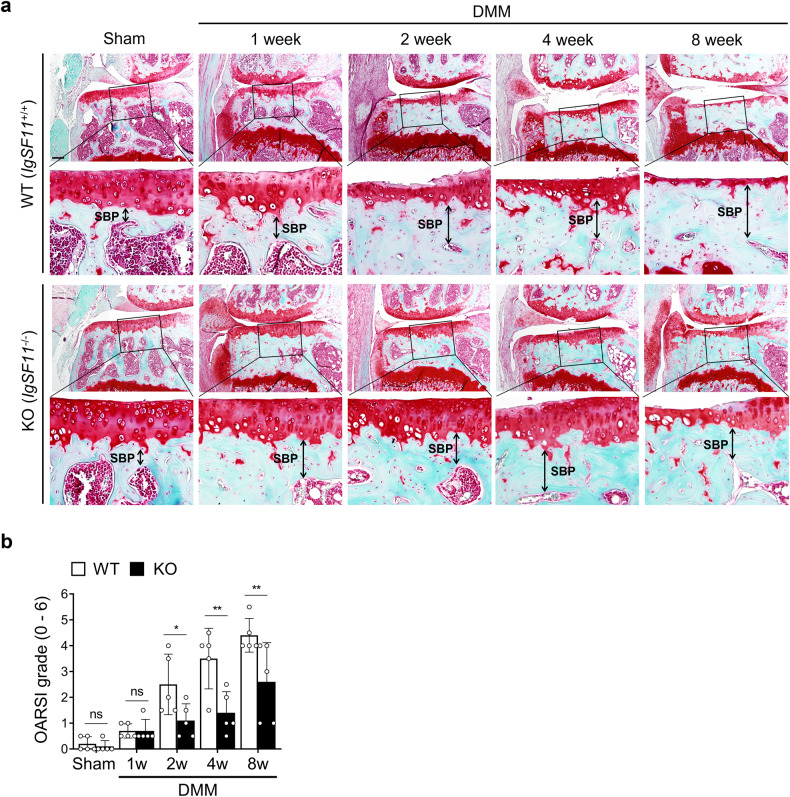
*IgSF11*^*−/−*^ mice exhibit reduced cartilage destruction after OA induction. **a**, **b** WT and *IgSF11*^*−/−*^ mice were subjected to DMM or sham surgery and euthanized at 1, 2, 4, or 8 weeks, and the joints were subjected to Safranin-O staining and fast-green counterstaining. **a** Representative images. SBP subchondral bone plate. The double-headed arrows indicate the thickening of the SBP in OA. Scale bars, 100 μm. **b** shows the OARSI scores. Error bars indicate the S.E.M. for *n* = 5/strain. Two-way ANOVA followed by Tukey’s multiple comparison test was conducted. *p* values are indicated in the figure (*p < 0.05, **p < 0.01).

To investigate the importance of IgSF11 in OA pathogenesis, we induced OA in WT and *IgSF11*^*−/−*^ mice with DMM surgery. To observe the gradual changes in articular cartilage and subchondral bone, we euthanized the mice at 1, 2, 4, or 8 weeks, and their joints were harvested. OARSI grading showed that the *IgSF11*^*−/−*^ mice exhibited significantly less articular cartilage destruction than the WT mice at 2, 4, and 8 weeks but not at 1 week (Fig. 2a, b). Thus, IgSF11 is needed for cartilage destruction in OA.

### *IgSF11*^*−/−*^ mice demonstrate less osteoclast activation in the SB soon after OA induction

Osteoclasts are key regulators of SB remodeling. They also interact with many cells in the joint, including osteoblasts, chondrocytes, and immune cells^8,11^. In particular, it is thought that while osteoblasts induce SB hardening in OA, this process is actually initiated by osteoclasts^24–26^. Since we found that (1) IgSF11 is upregulated in the SB soon after OA induction, (2) differentiating osteoclasts express IgSF11 in vitro, and (3) *IgSF11*^*−/−*^ mice demonstrate lower articular cartilage degradation throughout the course of OA, we speculated that IgSF11-expressing osteoclasts in the SB promote articular cartilage destruction in OA and that this role starts early.

**Fig. 3:**
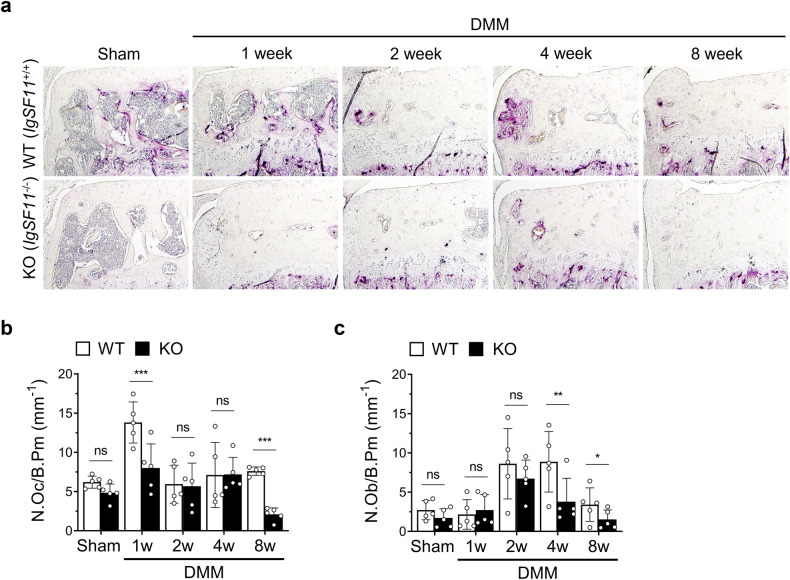
*IgSF11*^*−/−*^ mice show decreased osteoclast activation soon after OA induction. **a**–**c** DMM surgery-induced OA was generated in WT and *IgSF11*^*−/−*^ mice, and histological sections harvested at 1, 2, 4, or 8 weeks were subjected to TRAP staining. **a** Representative images of TRAP staining of mouse tibial subchondral bone. Scale bars, 200 μm. Histomorphometric analysis of the number of osteoclasts per bone perimeter (N.Oc/B. Pm) (**b**) and osteoblasts per bone perimeter (N.Ob/B. Pm) (**c**). Error bars show the S.E.M. for *n* = 5/strain. Two-way ANOVA followed by Tukey’s *t* tests was conducted. *p* values are indicated in the figures (*p < 0.05, **p < 0.01, ***p < 0.001).

To test this hypothesis, we examined the effect of *IgSF11* deletion on osteoclast and osteoblast activity during OA. Indeed, staining for the osteoclast marker TRAP showed that compared to WT mice, *IgSF11*^*−/−*^ mice exhibited significantly lower osteoclast activation at 1 week after DMM surgery (Fig. 3a, b). Moreover, compared to WT mice, *IgSF11*^*−/−*^ mice had fewer osteoblasts starting at 2 weeks (Fig. 3c). This finding supports the notion that IgSF11-expressing osteoclasts induce bone resorption soon after OA induction and that this process subsequently activates osteoblasts, which then form excessive bone.

### *IgSF11* knockout reduces SB changes during murine OA

**Fig. 4:**
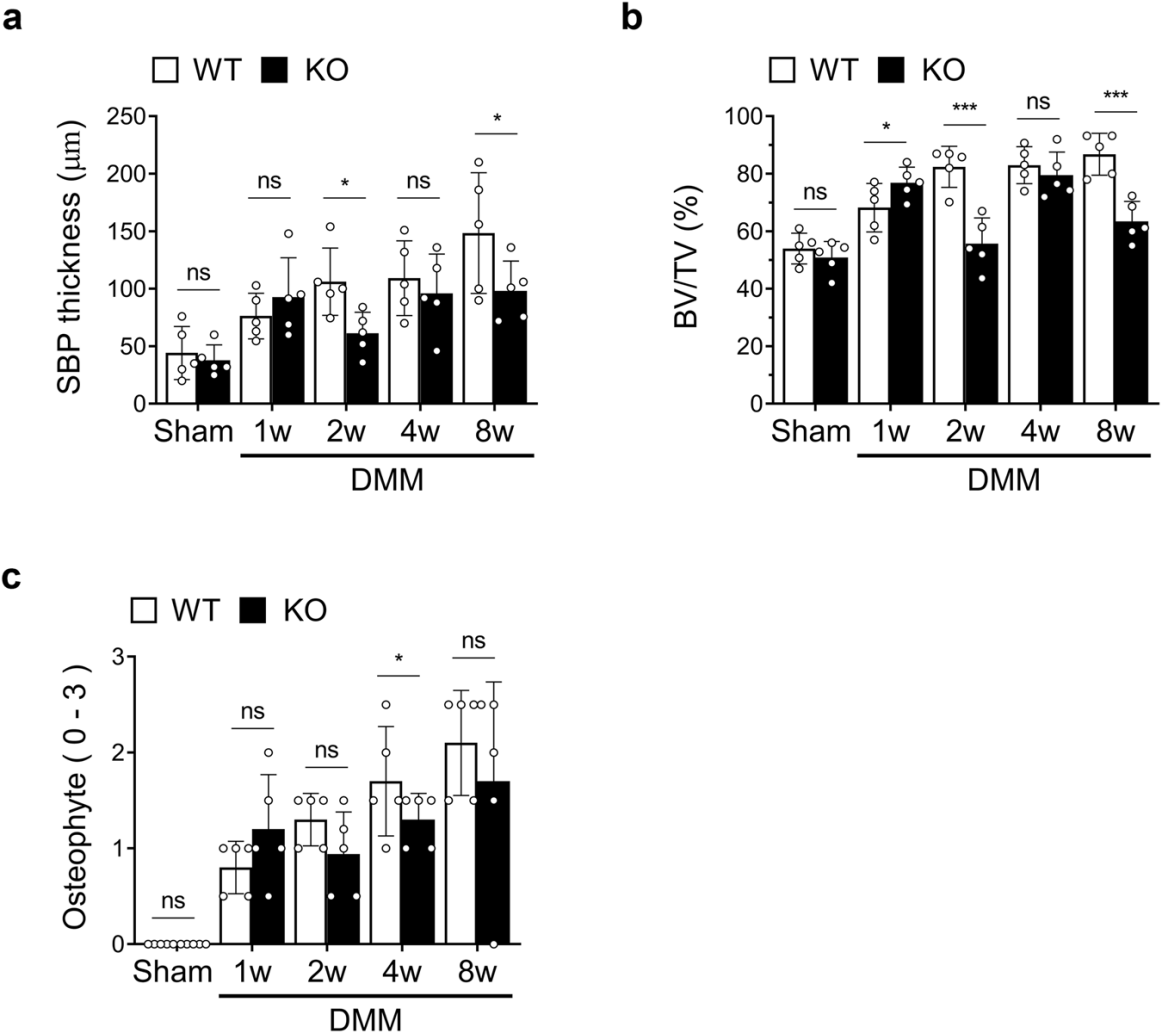
*IgSF11*^*−/−*^ mice display less subchondral bone thickening later in OA. DMM surgery-induced OA was generated in WT and *IgSF11*^*−/−*^ mice, and histological sections harvested at 1, 2, 4, or 8 weeks were subjected to histological scoring for subchondral bone variables in OA, namely, subchondral bone plate thickness (**a**), bone volume/trabecular volume (BV/TV) (**b**), and osteophyte maturation (**c**). Error bars show the S.E.M. for *n* = 5/strain. Two-way ANOVA followed by Tukey’s multiple comparison test was conducted. *p* values are indicated in the figures (*p < 0.05, ***p < 0.001).

The histological analyses shown in Fig. 2a indicates that while *IgSF11* deletion was associated with reduced SB plate (SBP) thickening later in OA, the SBP appeared to be thicker in *IgSF11*^*−/−*^ mice in the first week. To confirm these findings at a quantitative level, we measured SBP thickness with a histological scoring system for SB in OA^23,27^. We also measured two other key variables, namely, bone volume (BV/TV) and osteophyte maturation. Indeed, *IgSF11*^*−/−*^ mice demonstrated greater SBP thickening at 1 week, although this change did not achieve statistical significance. However, starting at 2 weeks, the knockout mice had significantly thinner SBP (Fig. 4a). Similar differences were observed for bone volume and osteophyte maturation (Fig. 4b, c). These differences are probably due to the low osteoclast activity in *IgSF11*^*−/−*^ mice: this phenomenon led to an osteoclast: osteoblast imbalance that led to initially more bone formation than bone resorption in the SBP. Later, however, the lack of lacunae meant that the osteoblasts were not activated and did not induce SBP thickening.

**Fig. 5:**
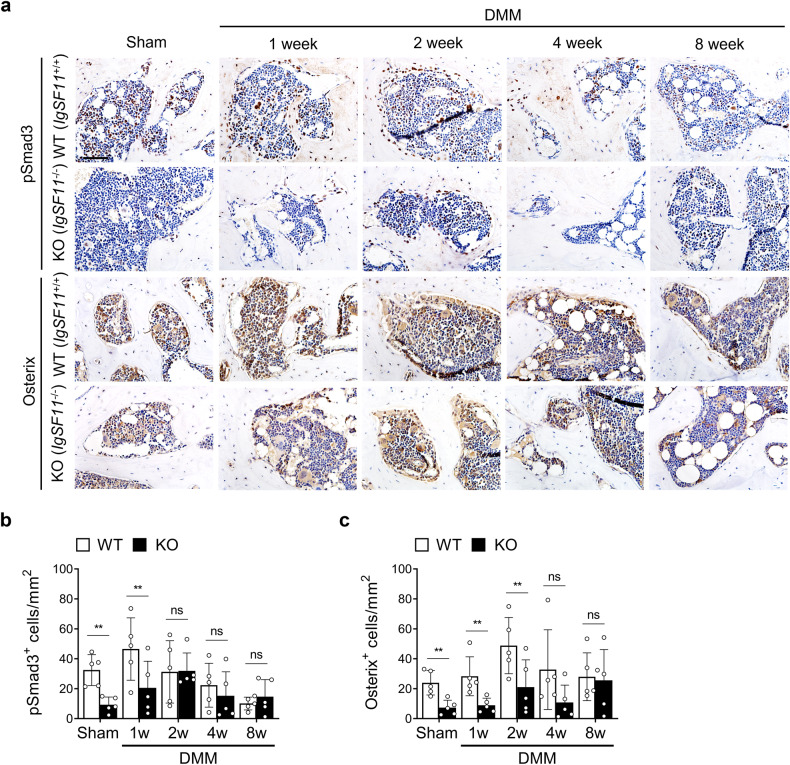
*IgSF11*^*−/−*^ mice exhibit reduced TGF-β signaling in osteoarthritic subchondral bone. **a**–**c** DMM surgery-induced OA was generated in WT and *IgSF11*^*−/−*^ mice, and histological sections harvested at 1, 2, 4, or 8 weeks were subjected to immunohistochemistry for pSmad3 and osterix. **a** Representative images of pSmad3^+^ cells (brown, top) and Osterix^+^ cells (brown, bottom) in mouse tibial subchondral bone. Scale bars, 200 µm. Quantitative analysis of the number of pSmad3^+^ (**b**) and Osterix^+^ (**c**) cells per bone marrow area (mm^2^). Error bars indicate the S.E.M. for *n* = 5/strain. Two-way ANOVA followed by Tukey’s *t* tests was conducted. *p* values are indicated in the figures (**p < 0.01).

The SB architectural changes in murine OA are associated with the release of active TGF-β by osteoclasts, which induces Smad2/3 phosphorylation in mesenchymal stem cells^28,29^. This process causes them to proliferate and differentiate into osterix^+^ osteoprogenitors, which accelerate aberrant bone formation and angiogenesis^28^. To determine whether *IgSF11* knockout interferes with this process, we conducted immunohistochemistry with anti-pSmad3 and anti-osterix. Indeed, both markers were decreased in the SB of *IgSF11*^*−/−*^ mice at 1 and 2 weeks (Fig. 5a–c). Thus, *IgSF11* knockout attenuates early SB changes in murine OA.

We confirmed the importance of IgSF11 in cartilage destruction in OA with another animal model of OA, namely, spontaneous age-induced OA. When we examined the articular cartilage and SB of 6- and 12-month-old WT and *IgSF11*^*−/−*^ mice (Supplementary Fig. 2a), we found that *IgSF11* deletion was associated with significantly less articular cartilage destruction at 12 (but not 6) months of age (Supplementary Fig. 2b). *IgSF11* knockout was also associated with greater SBP thickening and bone volume at 6 months but thinner SBP and smaller bone volume at 12 months (Supplementary Fig. 2c, d). In addition, we observed that as the WT mice started to develop OA at 6 months of age, their IgSF11 expression in the SB rose. This expression had normalized by the time OA was established at 12 months of age (Supplementary Fig. 2e). These findings are all similar to our observations with DMM surgery-induced OA (Figs. 2 and 4) and indicate that IgSF11 dictates both SB remodeling and articular cartilage destruction in not only post-traumatic OA but also age-associated OA.

### *IgSF11* knockout in osteoclasts reduces chondrocyte catabolism in OA

**Fig. 6:**
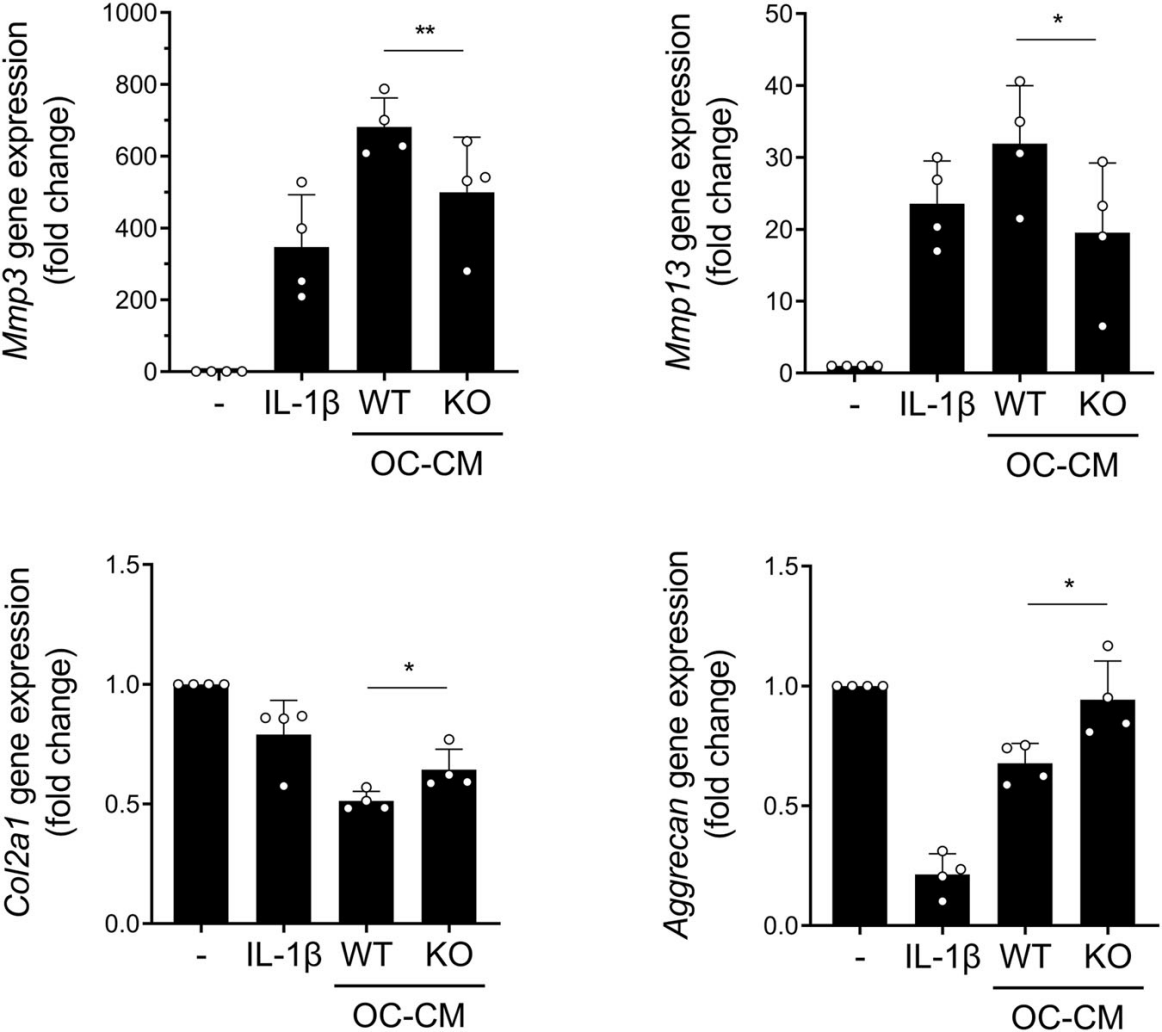
*IgSF11* deletion blocks the ability of the osteoclast secretome to enhance chondrocyte catabolism in vitro. BMMs from WT and *IgSF11*^−/−^ mice were induced to differentiate into mature osteoclasts on dentin slices with M-CSF and RANKL for 4 days. The OC-CM was collected on day 5. Primary articular chondrocytes isolated from naïve WT mice were treated for 24 h with the two CMs, after which they were subjected to qRT‒PCR for MMP3, MMP13, Col2A1, and Aggrecan expression. One-way ANOVA was performed followed by Dunnett’s multiple comparison test. *p* values are indicated in the figures (*p < 0.05, **p < 0.01).

The dynamic cellular and molecular interactions between SB and cartilage play important roles in joint homeostasis and OA development. In particular, the abnormal osteoclast formation and the resulting SB remodeling in OA are closely associated with increased catabolism and decreased anabolism of chondrocytes in the cartilage matrix^30,31^. The underlying mechanisms at least partly involve the release of bioactive molecules by osteoclasts^32,33^. Therefore, to test whether IgSF11 can mediate the ability of osteoclasts to shape chondrocyte catabolism/anabolism, we isolated osteoclast progenitor cells from naïve WT and *IgSF11*^*−/−*^ mice, induced them to differentiate into osteoclasts, harvested their conditioned medium (OC-CM), and then cultured WT primary articular chondrocytes with OC-CMs. As expected, WT CM markedly upregulated the mRNA expression of the matrix-degrading enzymes *Mmp3* and *Mmp13*^34,35^ in chondrocytes. In contrast, OC-CM from *IgSF11*^*−/−*^ osteoclasts showed weaker promotion of chondrocyte catabolism than WT OC-CM (Fig. 6). Thus, *IgSF11* knockout not only impaired the ability of osteoclasts to resorb bone but also weakened their release of molecules that increase chondrocyte catabolism.

To test this finding in vivo, we subjected the cartilage of WT and *IgSF11*^*−/−*^ mice with DMM surgery-induced OA to immunohistochemistry with antibodies against MMP3, MMP13, COL2A1, and Aggerecan. Indeed, the chondrocytes of WT mice increasingly expressed MMP3 and MMP13 as the disease progressed, and this effect was largely blocked when IgSF11 was knocked out (Supplementary Fig. 3). Conversely, WT chondrocytes expressed decreasing levels of COL2A1 and Aggrecan as the disease progressed, and this phenomenon was largely ameliorated in *IgSF11*^*−/−*^ mice (Supplementary Fig. 4). Thus, WT osteoclasts may produce molecules that stimulate chondrocyte catabolism, and this process depends on IgSF11. These findings together support the notion that IgSF11 promotes (1) osteoclast resorption and SB remodeling and (2) osteoclast-mediated regulation of chondrocytes and their destruction of cartilage in OA.

### IgSF11-expressing cells in the SB colocalize with RANK

**Fig. 7:**
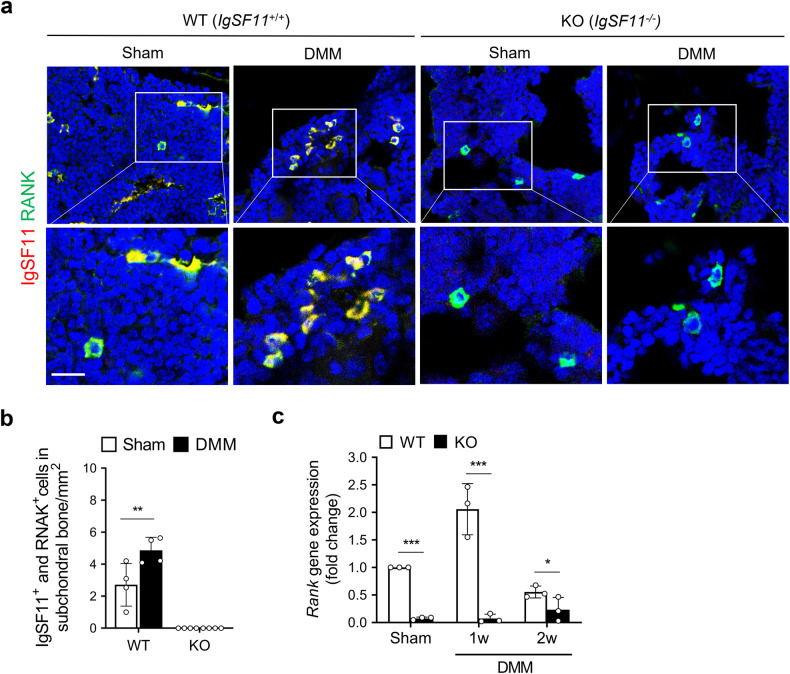
IgSF11-expressing cells in the subchondral bone of WT mice with OA colocalize with RANK. DMM surgery-induced OA was generated in WT and *IgSF11*^*−/−*^ mice. **a**, **b** Histological sections harvested at 1 week were subjected to double-immunofluorescence staining for IgSF11 and RANK. **a** Representative images of IgSF11^+^ cells (red) and RANK^+^ cells (green) and their colocalization (yellow) in mouse tibial subchondral bone. Scale bars, 20 μm. **b** Quantitative analysis of the number of IgSF11^+^RANK^+^ cells per subchondral bone marrow area (mm^2^). Error bars indicate the S.E.M. for *n* = 4/strain. Two-way ANOVA followed by Tukey’s *t* tests was conducted. **c** Subchondral bone tissue harvested at 1 or 2 weeks was subjected to qRT‒PCR for RANK. *p* values are indicated in the figures (*p < 0.05, **p < 0.01, ***p < 0.001).

To determine the precise location and morphology of the IgSF11-expressing cells in the SB of WT mice with DMM surgery-induced OA, we conducted double-immunofluorescence staining with IgSF11 and classic markers of osteoclast differentiation and function. These markers were RANK, which is a key osteoclast receptor that is required for osteoclast differentiation^36^; CSF-1R, which recognizes M-CSF and promotes osteoclast differentiation and maturation; TRAP, which is secreted by osteoclasts during bone resorption; CTSK, which is a key type-I collagen protease that is released by osteoclasts during bone resorption; and Atp6v0d2, which is an essential component of the osteoclast-specific proton pump that mediates extracellular acidification during bone resorption^36,37^. In WT mice, RANK colocalized with IgSF11-expressing cells 1 week after OA induction. These cells were mostly located in the bone marrow and had a round shape with one or two nuclei (Fig. 7a, b). However, CSF-1R, TRAP, CTSK, and Atp6v0d2 were mostly located on the SB surface and did not colocalize with IgSF11-expressing cells (Supplementary Fig. 5). Notably, qRT‒PCR showed that *IgSF11* knockout significantly decreased RANK mRNA expression in the SB of early OA (Fig. 7c). The bone marrow location of the RANK^+^IgSF11^+^ cells in the SB and their morphology suggest that the IgSF11-expressing cells are not mature osteoclasts. Nonetheless, given that IgSF11-expressing cells also express RANK, it is clear that IgSF11 is involved in osteoclast maturation and bone resorption in OA.

## Discussion

Decades of basic and clinical research on OA have shown that this disease is characterized by articular cartilage destruction. However, therapeutic strategies that target this feature have limited efficacy. This issue may be because the more important structure in OA development and progression is the “osteochondral unit”, namely, the interactive complex that consists of cartilage and the underlying SB. This hypothesis is supported by a growing body of research showing that the SB plays a key role in articular cartilage structure and function. Specifically, articular cartilage is aneural, avascular, and alymphatic and thus depends heavily on the SB for nutritional and mechanical support^8,11^. Indeed, during OA development, the SB displays microstructural and pathological changes before articular cartilage changes manifest themselves. Thus, targeting SB remodeling in OA or the interactions between the SB and cartilage may have better clinical outcomes. A target that may be of particular interest is the osteoclast. While SB remodeling involves many dynamically interactive cell types in the SB, including osteocytes, osteoblasts, osteoclasts, endothelial cells, and sensory neurons^12^, it is likely that osteoclasts play a particularly central early role in OA: these cells orchestrate SB remodeling and mediate local angiogenesis and sensory nerve innervation^11,24,25^, and blocking osteoclast functions soon after OA induction in murine models attenuates SB remodeling, cartilage destruction, and pain^38–40^.

To help increase the treatment options for OA, we investigated the role of IgSF11 in OA. We found that IgSF11 is quickly expressed by BMMs after osteoclastogenic stimulation in vitro, which confirms that it is required for osteoclast maturation^19,20^. Moreover, this molecule is upregulated in RANK-coexpressing osteoclast-like cells in the SB at 1 week after OA induction. Importantly, *IgSF11* knockout in mice with OA significantly (1) reduced osteoclast activation, TGF-β/pSMAD2/3-induced MSC activation, and osterix^+^ osteoprogenitor numbers in the SB in vivo; (2) decreased SB osteoblast numbers and reduced SBP thickening starting at 2 weeks; and (3) reduced catabolism of the articular cartilage starting at 2 weeks, thus reducing cartilage degradation in OA.

Interestingly, the RANK^+^IgSF11^+^ cells that emerged in the SB 1 week after OA induction were round and located in the bone marrow. These cells are likely to be osteomorphs, which are the daughter cells generated from osteoclast fission. Osteomorphs were identified only recently, and their specific cell-surface markers remain to be defined^41^. Very little is known about them except that osteoclasts not only produce osteomorphs but also fuse to create large osteoclasts. This process is termed osteoclast recycling. Both fission and fusion events are driven by RANKL, and the whole process regulates bone resorption because the osteoclasts generated by osteomorph fusion are highly active^41,42^. Notably, comparative transcriptomics showed that compared to macrophage precursors, 151 genes were upregulated in osteomorphs but not in osteoclasts. Of these 151 genes, 17 are related to bone structure or function, and two are Vcam1 and Cadm1^41^. Like IgSF11, these are immunoglobulin superfamily members that function as cell adhesion molecules^43–45^. These molecules could play key roles in osteomorph fusion since cell-adhesion molecules are known to mediate cell fusion in other settings. Indeed, IgSF11 has a highly similar function, namely, it is responsible for the cell fusion involved in osteoclast differentiation^19,41^. While it is not yet known whether IgSF11 is expressed by osteomorphs, our study supports this hypothesis. These intriguing observations suggest that further studies on the roles of SB osteomorphs in health and OA and whether these roles are mediated by IgSF11 are warranted.

### Supplementary information

Supplementary Information
