# Supplementary material for: IgSF11 deficiency alleviates osteoarthritis in mice by suppressing early subchondral bone changes
Source: Exp Mol Med. 2023 Dec 1;55(12):2576–85. doi: 10.1038/s12276-023-01126-6 (PMC10767117; doi:10.1038/s12276-023-01126-6)
Supplement: Supplementary file 1 — Supplementary Information [file 12276_2023_1126_MOESM1_ESM.pdf]

Supplemental Information

IgSF11 deficiency alleviates osteoarthritis in mice by suppressing early subchondral bone changes

(Running title: The role of IgSF11 in osteoarthritis pathogenesis)

Gyeong Min Kim[1,2], Jihee Kim[1,2], June-Yong Lee[3], Min-Chan Park[4], and Soo Young Lee[1,2,5*]

[1]Department of Life Sciences, Ewha Womans University, Seoul 03760, Republic of Korea
[2]The Research Center for Cellular Homeostasis, Ewha Womans University, Seoul 03760, Republic of Korea
[3]Department of Microbiology and Immunology, Yonsei University College of Medicine, Seoul 03722, Republic of Korea
[4]Division of Rheumatology, Department of Internal Medicine, Yonsei University College of Medicine, Seoul 06273, Republic of Korea
[5]Multitasking Macrophage Research Center, Ewha Womans University, Seoul 03760, Republic of Korea

*Correspondence should be addressed to:

S.Y. Lee, Department of Life Sciences, Ewha Womans University, Seoul 03760, South Korea.

Tel: 82-2-3277-3770; Fax: 82-2-3277-3760; E-mail: leesy@ewha.ac.kr

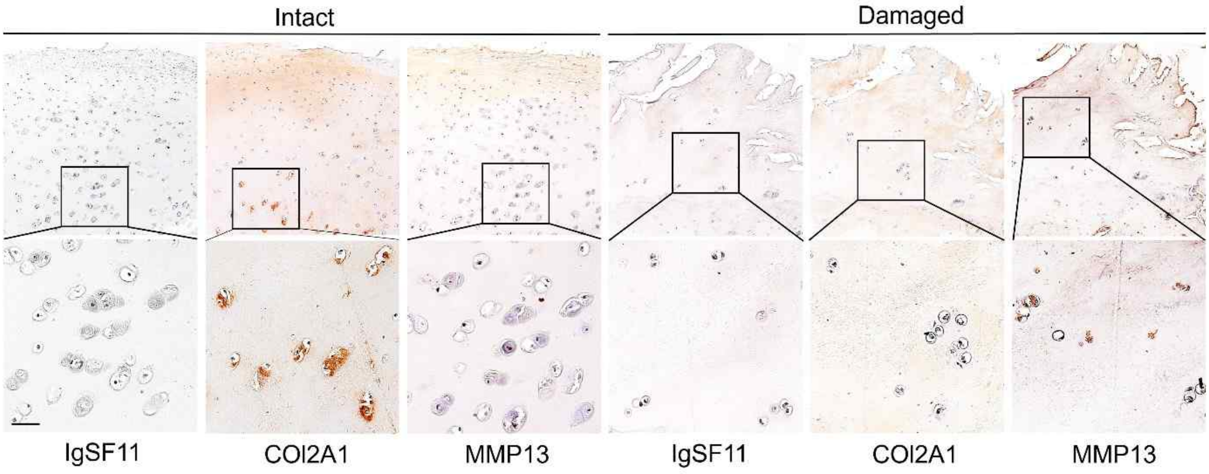

Supplementary Fig. 1. IgSF11 is not expressed in the damaged or undamaged cartilage of patients with OA.

Representative images of the intact and damaged regions of articular cartilage from OA patients after immunohistochemical staining for IgSF11, COL2a1, and MMP13. Scale bars, 100 μm.

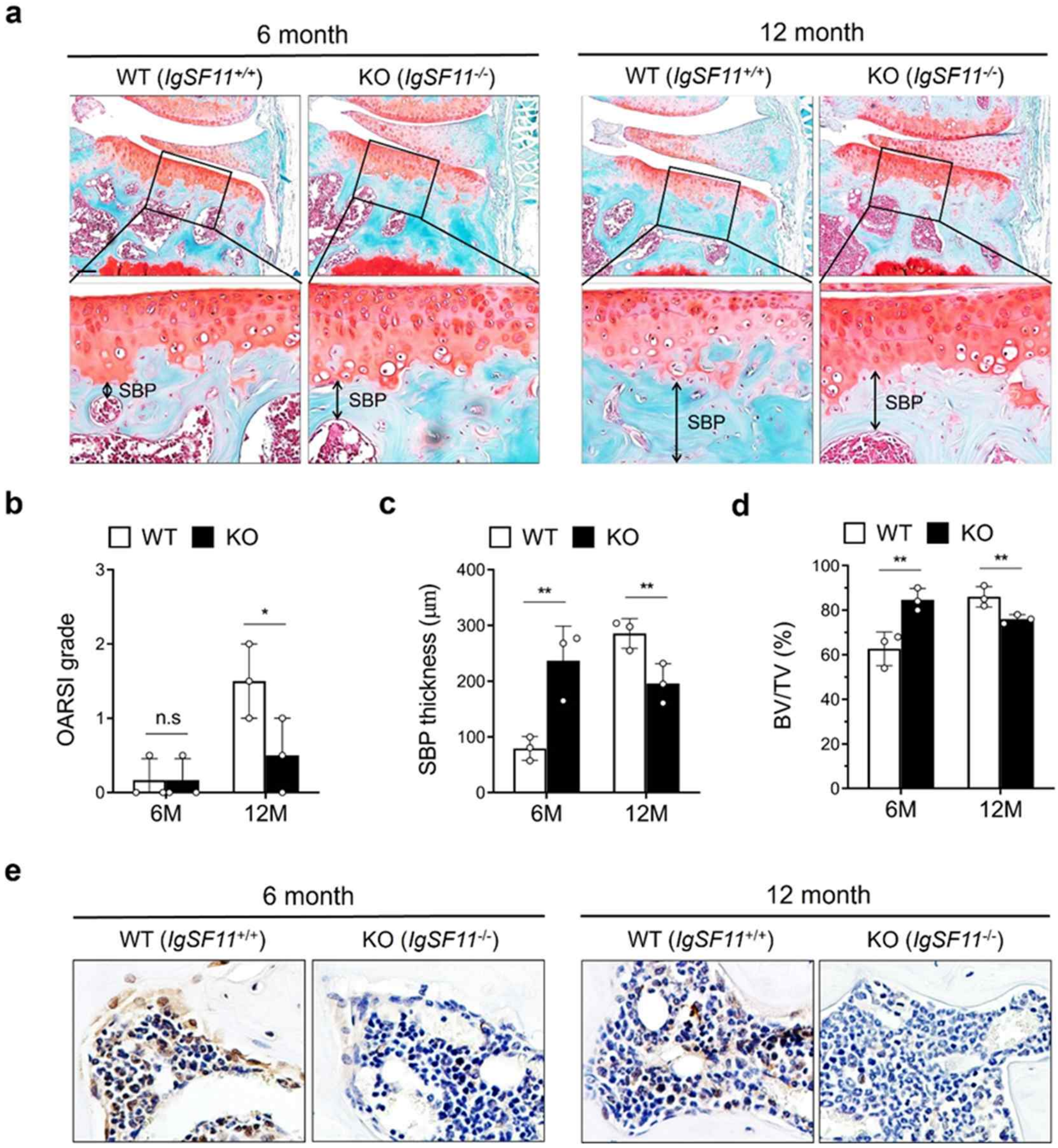

Supplementary Fig. 2. ***IgSF11*** **knockout decreases articular cartilage destruction subchondral bone remodeling in age-associated OA.**

a-e, WT and Ig*SF11*$^{-/-}$ mice were euthanized at 6 or 12 months of age and the joints were subjected to Safranin-O staining and fast-green counterstaining. (**a**) shows representative images. The double-headed arrows indicate the thickening of the subchondral bone-plate (SBP). Scale bars, 100 μm. (**b**) OARSI score. (**c**) SBP thickness. (**d**) Bone volume/trabecular volume (BV/TV). (**e**) Representative images of IgSF11^{+} cells (brown) in mouse subchondral bone. Error bars indicate S.E.M. for n=3/strain. Two-way ANOVA followed by Tukey's Multiple comparison's test was conducted. P-values are indicated in the figure (* $p < 0.05$, ** $p < 0.01$).

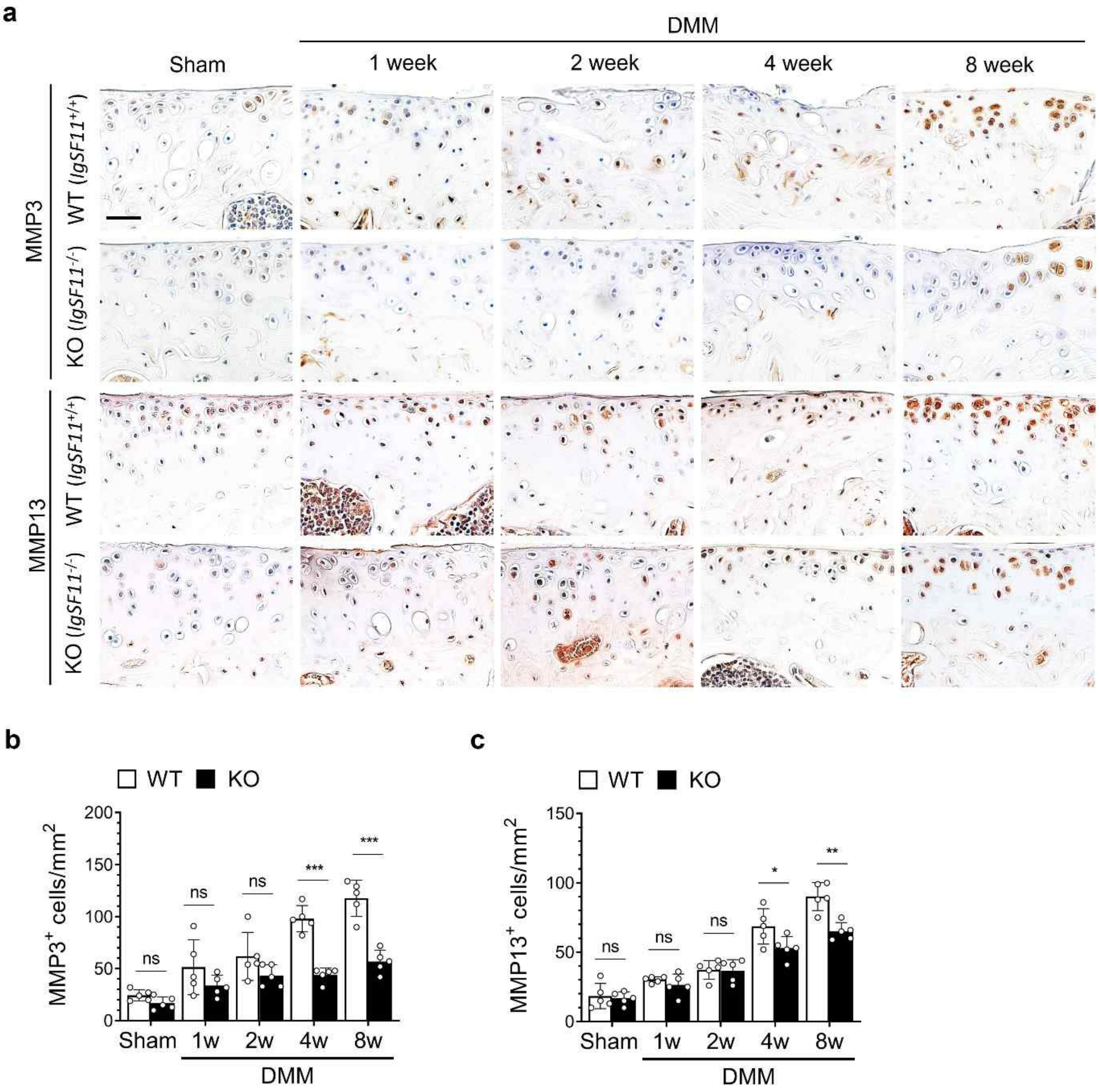

Supplementary Fig. 3. *IgSF11*$^{-/-}$ mice show decreased cartilage-matrix catabolism in OA.

a-c, DMM surgery-induced OA was generated in WT and *IgSF11*$^{-/-}$ mice and histological sections harvested at 1, 2, 4, or 8 weeks were subjected to immunohistochemistry for MMP3 and MMP13. (**a**) Representative images of MMP3$^+$ cells (brown, top) and MMP13$^+$ cells (brown, bottom) in the cartilage. Scale bars, 20 µm. (**b,c**) Quantitative analysis of the number of MMP3$^+$ (**b**) and MMP13$^+$ (**c**) cells per bone-marrow area (mm^2). Error bars indicate the S.E.M. for n=5/strain. Two-way ANOVA followed by Tukey's t tests was conducted. P-values are indicated in the figures ($* \ p < 0.05$, $** \ p < 0.01$, $*** \ p < 0.001$).

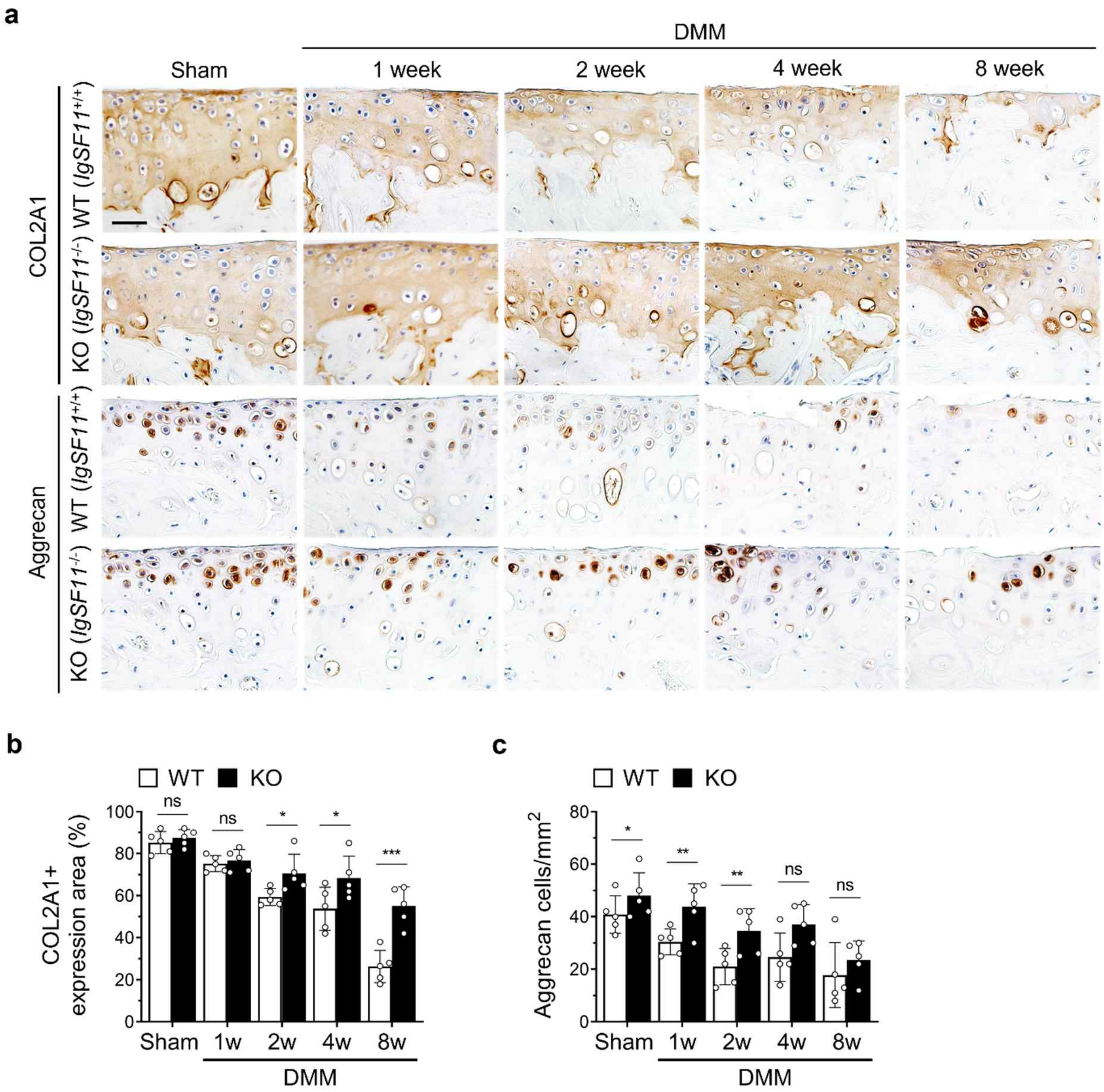

Supplementary Fig. 4. ***IgSF11*$^{-/-}$ mice show increased cartilage-matrix anabolism.**

a-c, DMM surgery-induced OA was generated in WT and *IgSF11*$^{-/-}$ mice and histological sections harvested at 1, 2, 4, or 8 weeks were subjected to immunohistochemistry for Col2A1 and Aggrecan. (**a**) Representative images of Col2A1^{+} cells (brown, top) and Aggrecan^{+} cells (brown, bottom) in mouse cartilage. Scale bars, 20 µm. (**b,c**) Quantitative analysis of the number of Col2A1^{+} (**b**) and Aggrecan^{+} (**c**) cells per bone-marrow area (mm^2). Error bars indicate the S.E.M. for n=5/strain. Two-way ANOVA followed by Tukey's t tests was conducted. P-values are indicated in the figures (* $p < 0.05$, ** $p < 0.01$, *** $p < 0.001$).

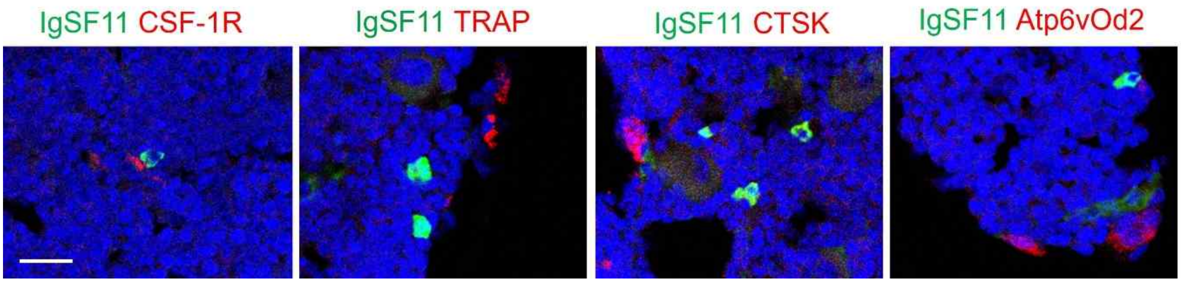

Supplementary Fig. 5. IgSF11 does not colocalize with matured osteoclast markers. DMM surgery-induced OA was generated in WT mice and histological sections harvested at 1 week were subjected to double-immunofluorescence staining for IgSF11 (green) and CSF-1R, TRAP, CTSK, or Atp6vOd2 (all red). Scale bars, 20 μm.
